# Visual and Proprioceptive Perceptions Evoke Motion-Sound Symbolism: Different Acceleration Profiles Are Associated With Different Types of Consonants

**DOI:** 10.3389/fpsyg.2020.589797

**Published:** 2020-11-12

**Authors:** Kazuko Shinohara, Shigeto Kawahara, Hideyuki Tanaka

**Affiliations:** ^1^Language and Culture Studies, Tokyo University of Agriculture and Technology, Tokyo, Japan; ^2^The Institute of Cultural and Linguistic Studies, Keio University, Tokyo, Japan; ^3^Human Movement Science, Tokyo University of Agriculture and Technology, Tokyo, Japan

**Keywords:** cross-modal correspondence, non-arbitrariness, passive movement, obstruents, sonorants

## Abstract

A growing body of literature has shown that one perceptual modality can be systematically associated with sensation in another. However, the cross-modal relationship between linguistic sounds and motions (i.e., motion-sound symbolism) is an extremely understudied area of research. Against this background, this paper examines the cross-modal correspondences between categories of consonants on one hand and different acceleration profiles of motion stimuli on the other. In the two experiments that we conducted, we mechanically manipulated the acceleration profiles of the stimuli while holding the trajectory paths constant, thus distinguishing the effect of acceleration profiles from that of motion path shapes. The results show that different acceleration profiles can be associated with different types of consonants; in particular, movements with acceleration and deceleration tend to be associated with a class of sounds called obstruents, whereas movements without much acceleration tend to be associated with a class of sounds called sonorants. Moreover, the current experiments show that this sort of cross-modal correspondence arises even when the stimuli are not presented visually, namely, when the participants’ hands were moved passively by a manipulandum. In conclusion, the present study adds an additional piece of evidence demonstrating that bodily action-based information, i.e., proprioception as a very feasible candidate, could lead to sound symbolic patterns.

## Introduction

We have recently witnessed a tremendous increase of interest in studies of cross-modal perception, in which sensation in one modality is systematically associated with sensation in another (e.g., Spence, 2011). Particular cases of such cross-modal perception involving linguistic sounds have been extensively studied under the general rubric of “sound symbolism,” in which some linguistic sounds are associated with certain meanings and images, such as size, shape, color, hardness, and weight (Jesperson, 1922; Sapir, 1929; Hinton et al., 1994; Nuckolls, 1999; Berlin, 2006; Akita, 2015; Dingemanse et al., 2015; Lockwood and Dingemanse, 2015).

The very existence of sound symbolism challenges the notion of the arbitrariness of linguistic signs (de Saussure, 1916)–arbitrary connections between sounds and meanings–which has been accepted as one of the fundamental principles of modern linguistics theories in the 20th century. The arbitrary nature of linguistic signs has long been considered to be a crucial property of human language that distinguishes it from other animals’ communication systems (Hockett, 1959). However, the arbitrariness thesis is no longer a principle that is thought to hold without exceptions to the degree that several sound symbolic patterns hold ubiquitously across different languages. Moreover, the sound symbolic associations may instantiate *embodied* motivation in human languages, one of the fundamental tenets of cognitive linguistics (Lakoff and Johnson, 1980, 1999; Lakoff, 1987). That is, our bodily experiences may impact and shape the linguistic structures of human languages. Sound symbolic patterns instantiate embodied motivations because in most sound symbolic patterns, how we speak and perceive sounds are iconically mapped onto the meaning of these sounds (Perniss and Vigliocco, 2014). There is thus a rise of interest in studies of sound symbolism among linguists, especially cognitive linguists (e.g., Akita, 2009).

One well-studied case of sound symbolism is size-related sound symbolism. Sapir’s pioneering study (Sapir, 1929) demonstrated, for example, that the high front vowel [i] tends to be judged to be smaller than the low back vowel [a]. The size-related sound symbolism has been shown to hold for speakers of English (Sapir, 1929; Ohala, 1994; Thompson and Estes, 2011), Japanese, Mandarin, and Korean (Shinohara and Kawahara, 2016) and Cantonese and Russian (Shih et al., 2019). It seems safe to conclude from this ever-increasing body of studies that size-related sound symbolism is a general property of human languages.

**FIGURE 1:**
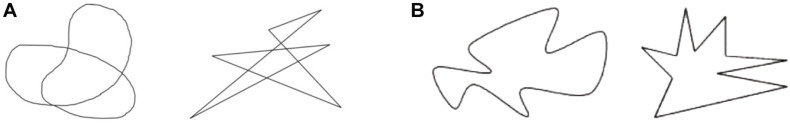
**(A)** Köhler’s figures: *maluma* (left) and *takete* (right); **(B)** Ramachandran’s and Hubbard’s figures: *bouba* (left) and *kiki* (right).

Another case of sound symbolism that has been extensively studied is shape-related sound symbolism. A well-known example is the case discussed by Köhler (1929). Köhler (1947) argued that the nonce word *takete* is likely to be associated with an angular/spiky shape, whereas the nonce word *maluma* tends to be associated with a round/curvy shape (Figure 1A). More recently, Ramachandran and Hubbard (2001) demonstrated that most people associate a round shape with the nonce word *bouba*, while they associate an angular shape with *kiki* (Figure 1B). A large body of follow-up research has found that these kinds of shape-sound associations hold robustly across speakers of many different languages (Irwin and Newland, 1940; Holland and Wertheimer, 1964; Kim, 1977; Lindauer, 1990; Berlin, 2006; Ahlner and Zlatev, 2010; Nielsen and Rendall, 2011, 2013; Kawahara and Shinohara, 2012, 2015; Bremner et al., 2013; Haynie et al., 2014; Nobile, 2015; Blasi et al., 2016; Styles and Gawne, 2017, among others)^1^. Moreover, sound symbolic patterns have been shown to hold among infants (Ozturk et al., 2012), toddlers (Maurer et al., 2006), and even neonates (Peña et al., 2011). Therefore, it seems safe to conclude that humans can associate certain perceptual properties with certain linguistic sounds.

Size and shape are two major topics of exploration on sound symbolism (Sidhu and Pexman, 2017, 2019). In addition to size and shape, there are other perceptual dimensions that have been demonstrated to participate in sound symbolic associations, which include, but are not limited to, hardness, weight, brightness, and even personal characteristics (Uemura, 1965; Kawahara et al., 2018; Sidhu and Pexman, 2019; Uno et al., 2020). However, in most previous studies of sound symbolism, the focus has been exclusively on the five senses (visual, auditory, tactile, olfactory, and gustatory), with a heavy emphasis on visual properties, such as size and shape. While there is little doubt that previous studies on sound symbolism have provided important insights into human cognitive capacities, some issues remain underexplored. To our knowledge, only a few experimental studies have explored sound symbolic associations with dynamic motions (Cuskley, 2013; Koppensteiner et al., 2016; Shinohara et al., 2016).

Motion is an emerging topic in sound symbolism studies. A few previous studies that explored sound-symbolic associations with dynamic motion stimuli have primarily focused on visual perception. For instance, Cuskley (2013) used bouncing ball stimuli to show that reduplication, voicing, and vowel quality are sound-symbolically related to the visual perception of motion velocity. More recently, Koppensteiner et al. (2016) demonstrated that visual perception of dynamic gestures and consonant sounds can have the same sound-symbolic associations as the case of static round vs. angular shapes. Shinohara et al. (2016) also employed visual stimuli of dynamic gestures tracing round vs. angular shapes to demonstrate that obstruent consonants tend to be associated with angular motions while sonorant consonants tend to be associated with round motions. All of these studies have demonstrated that dynamic motions can have sound-symbolic effects, showing that specific kinematic features of moving objects, such as trajectory, amplitude, and velocity, can be mapped onto particular types of sounds. However, motion properties perceived via modalities other than vision, especially proprioceptive perception (i.e., perception of stimuli relating to body position, posture, equilibrium or movement), are heavily understudied. The present study addresses these gaps in the literature by exploring how visual and proprioceptive stimuli can be symbolically mapped onto linguistic sounds. Particularly, our experimental study addresses the question of whether different types of acceleration profiles can be systematically associated with particular sets of sounds. The present study answers this question positively, thereby expanding the scope of known cases of cross-modal correspondences. We contend that this is an important finding, not only because this is a topic that has never been addressed before, but also because motion-related sound symbolic associations may have played an important role in the origin and evolution of human languages (Larsson, 2015) and may also play a non-trivial role in language acquisition (Imai and Kita, 2014). Furthermore, since proprioception provides internal representations of one’s own body movements, including articulatory gestures of the mouth and tongue, our current challenge in exploring the motion-sound symbolism in proprioceptive perception can facilitate the attainment of a more in-depth understanding of how our bodily experiences impact and shape the linguistic structures of human languages (Lakoff and Johnson, 1980, 1999; Lakoff, 1987).

There are three reasons for the examination of the possible sound symbolic nature of dynamic movements. First, sound symbolism related to dynamic motion has been suggested to play a key role in the origin and evolution of human languages. Larsson (2015) argued that “[t]he production and perception of sound, particularly of incidental sound of locomotion and tool-use sound (TUS), also contributed to the evolution of language” and that “[s]ince the human brain proficiently extracts information about objects and events from the sounds they produce, TUS, and mimicry of TUS, might have achieved an iconic function.” Larsson (2015) further suggested that this idea is supported by the prevalence of sound symbolism in many extant languages. In fact, people can relate not only TUS but also sounds produced by our bodily movements with linguistic sounds. For instance, Margiotoudi and Pulvermüller (2020) provided an experimental evidence that action-based shape-sound mappings (e.g., figure shapes drawn by a pen and sounds produced by the movement of the pen) correlate with associations in shape-sound symbolism. They argued that the sound symbolic knowledge that interlinks speech sounds and abstract shapes can arise from a tripartite interaction between action-visual-auditory information immanent to action experience, along with acoustic similarities between speech and action sounds as well as visual similarities between visually perceived actions and classic sound symbolic shapes. They suggested that the action-based shape-sound mappings can easily be learned when observing one’s self or another person acting or otherwise producing such shapes. Unlike figures drawn by a pen, however, the shape of bodily motion does not always remain as a physical, visible object. Meanwhile, one’s own bodily movements are more readily coded by both vision and proprioception, that is, as kinematic properties such as position, speed, and change of speed. Such kinematic information may be more salient than information consisting only of the shapes of motion trajectory. Thus, building upon Margiotoudi’s and Pulvermüller’s (2020) findings, the present study explores further the effects of acceleration patterns of motion, particularly focusing on proprioceptive perception of one’s own bodily motion. By keeping the shapes of motion paths constant, we test whether acceleration and deceleration, i.e., changes in speed, can override the abstract shapes of motion. Thus, our study is the first attempt to incorporate the sensorimotor domain of dynamic bodily movements, i.e., proprioceptive perception, in the study of sound symbolism, thereby providing certain credibility to Larsson’s proposal about language evolution from the kinematics perspective of motion-sound symbolism.

Second, our study aims, albeit indirectly, to contribute to the body of research that explores the possible role of sound symbolism in language acquisition processes. A series of studies have demonstrated that sound-symbolic words, including mimetics/onomatopoetic words, can facilitate language learning (Imai et al., 2008, 2015; Kantartzis, 2011; Kantartzis et al., 2011; Imai and Kita, 2014; Asano et al., 2015). These studies generally relied upon complex action stimuli that were sound-symbolically matched with particular mimetic words, e.g., a rabbit moving in a particular fashion. These stimuli, however, do not allow us to pin down precisely which aspects of the motion triggered the sound-symbolic patterns at issue. Investigating motion-sound symbolism using mechanically controlled stimuli may, therefore, have the potential to complement the results of these previous studies on the impact of sound symbolism on language acquisition.

Third, studying sound symbolism related to dynamic motion and proprioceptive perception of one’s own bodily motion can offer insights that can be useful in other academic fields, such as sports, physical therapy, and rehabilitation sciences. For instance, we may be able to bear upon the mechanism by which visual and proprioceptive representations of bodily movements can be communicated verbally between athletes and coaches (Yamauchi et al., 2016, 2019). In addition, investigating sound symbolism with a special focus on movements and kinematic properties can be relevant for a sector of food science that investigates the associations between linguistic sounds and the experience of eating foods–including the perception of food texture and taste and that of the physical activity of biting (Zampini and Spence, 2004; Dijksterhuis et al., 2007; Gallace et al., 2011; Spence, 2015; Sakamoto and Watanabe, 2016; Winter et al., 2019).

With these general issues in mind, the present study zooms in on the associations between dynamic motions and linguistic sounds as its empirical domain. Two experiments were conducted to address whether sound symbolic associations with particular types of motions can be identified in two different modalities: vision and proprioception. We used mechanically controlled motion stimuli based on a computational algorithm to pin down the key kinematic elements that can evoke sound-symbolic associations.

The present study explores how both visual perception and proprioceptive perception of dynamic motions connect with consonant sounds. Based on previous findings (Koppensteiner et al., 2016; Shinohara et al., 2016), we predict that such a dynamic motion-consonant association would appear in both modalities. The linguistic distinction that we are primarily interested in is the distinction between obstruents (consonants that involve a substantial rise in intraoral air pressure, including plosives, affricates, and fricatives, such as /p/, /t/, /k/, /b/, /d/, /g/, /s/, and /z/) and sonorants (consonants that involve little rise in intraoral air pressure, including nasal, liquids, and glides, such as /m/, /n/, /r/, /l/, /j/, and /w/). We tested whether these two sound categories are associated with particular patterns of kinematic properties, more specifically, two types of acceleration. One is a movement involving clear acceleration and deceleration (henceforth, ACC). The other is a movement with no tangible acceleration or deceleration (henceforth, CNST). Our hypothesis is as follows:

**Hypothesis:** Obstruents are more likely to be associated with ACC motions, while sonorants are more likely to be associated with CNST motions.

This hypothesis was motivated by the insights obtained from previous studies that examined the associations between dynamic motions and linguistic sounds. Köhler’s work (Köhler, 1929, 1947), as well as related subsequent studies, examined static visual shapes, showing that obstruents tend to be associated with statistic angular shapes, whereas sonorants tend to be associated with round shapes. Moreover, Koppensteiner et al. (2016) and Shinohara et al. (2016) demonstrated that dynamic movements that involve angular trajectories (like the *takete* figure) tend to be associated with obstruents, whereas dynamic movements with smooth trajectories (like the *maluma* figure) tend to be associated with sonorants.

**FIGURE 2:**
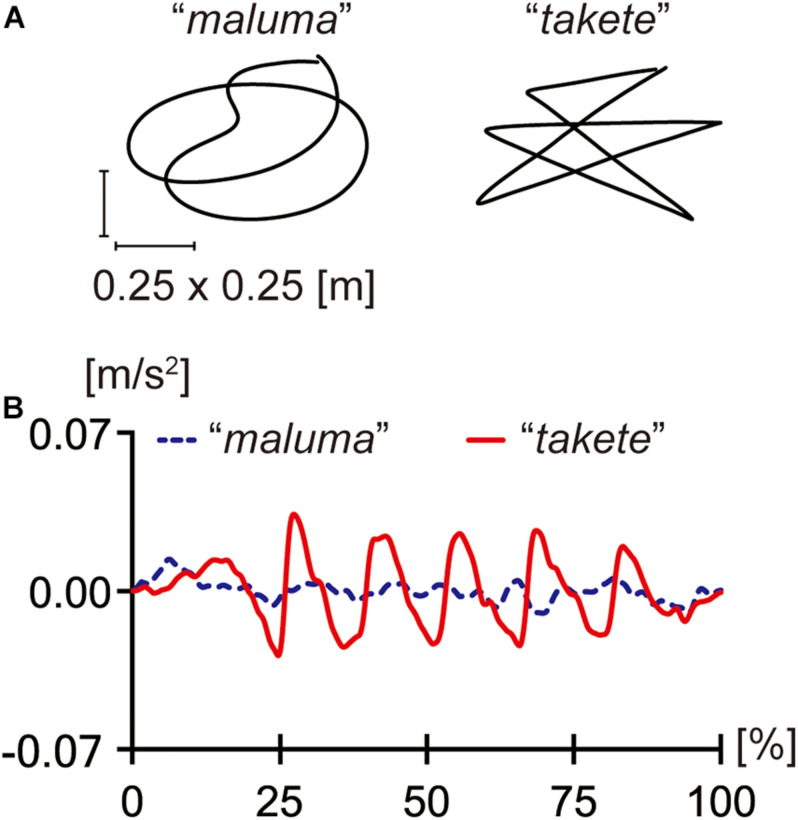
The trajectories **(A)** and acceleration profiles **(B)** of the *maluma* and *takete* movements. The *x*-axis in **(B)** shows the normalized time course of each movement in percentages. The figures were adapted from Shinohara et al. (2016) with slight edits.

These previous studies distinguished two types of movements: one that moves along an angular path and another that moves along a round path (Koppensteiner et al., 2016; Shinohara et al., 2016). However, they did not distinguish the shapes of trajectories from the differences in acceleration profiles. As Figure 2 shows, the two trajectories used by Shinohara et al. (2016) had different acceleration profiles. The trajectory tracing the *maluma* figure had a more or less constant speed with no tangible acceleration, while the trajectory tracing the *takete* figure showed clear acceleration and deceleration.

The present study thus addressed the question of whether different acceleration profiles can be associated with certain sound types independent of trajectory paths. To this end, we prepared mechanically controlled motion stimuli in which acceleration profiles differed systematically, while the trajectories were kept constant.

## Materials and Methods

### Outline of the Method

The current study had two conditions: (a) a movement with clear acceleration and deceleration (ACC) and (b) a movement with no tangible acceleration or deceleration (CNST). These two movements followed exactly the same round path^2^. Two experiments were conducted. Experiment 1 tested whether different acceleration patterns in the *visual perception* of dynamic movements can result in sound symbolic associations regarding the obstruent/sonorant distinction. Experiment 2 tested whether different acceleration patterns in the *proprioceptive perception* of one’s own bodily motion can result in different sound symbolic associations. In Experiment 1, the participants were visually presented with moving stimuli with different acceleration profiles (ACC and CNST), while in Experiment 2, the participants experienced their hands moved passively with the two acceleration profiles (ACC and CNST) using a robotic manipulandum.

The procedures employed in the two experiments were the same, except for the differences in perceptual stimuli inputs. All of the participants were native speakers of Japanese who were naïve as to the purpose of the experiments. All procedures in the two aforementioned experiments were approved by the ethics review board of the first author’s institution (Permit No. 27-04).

The requisite sample size of 84 participants (42 participants for each of the two experiments) was determined using the G^∗^Power program (version 3.1, Düsseldorf, Germany; Faul et al., 2007) for a repeated-measures test (within-participant factor consisting of two measurements: ACC and CNST conditions) based on α = 0.05, power (1–β) = 0.95, and effect size *f* = 0.58, which was directly calculated by partial eta-squared (η_*p*_^2^) = 0.25 (i.e., large effect size).

### Task and Measurements

In both Experiments 1 and 2, a free name elicitation task was employed, as adopted from Berlin (2006). This method was employed in order to overcome possible drawbacks of a forced-choice method, a strategy that is commonly deployed in experiments on sound symbolism. Early experimental work on the *takete-maluma* effects often relied upon a small number of linguistic stimuli. In such studies, the experimental manipulations could have been made clear to the participants (Westbury, 2005; Westbury et al., 2018). Moreover, as Westbury (2005) pointed out, “[t]he sound symbolism effects may depend largely on the experimenter pre-selecting a few stimuli that he/she recognizes as illustrating the effects of interests” (p. 11).

To avoid these potential problems, the present study employed a free name elicitation task in which the participants were asked to come up with novel words that would match each stimulus. While some restrictions were imposed on the syllable structures and the kinds of phonemes that can be used, the participants were free to come up with new names.

At the beginning of the experiments, the participants were asked to imagine that they were assistants of Steven Spielberg, helping him prepare for his new science fiction film. They were told that in that film, Droids, a kind of fictional robot in a remote planet, spoke a language called Droidese, a language that was different from the participants’ native language or any language they knew. The participants were asked to come up with novel words in Droidese, which they felt to be suitable for each of the experimental stimuli. The participants were told that Droidese had the following sound system:

**The sound inventory of Droidese:**

• Consonants: /p/, /t/, /k/, /b/, /d/, /g/, /s/, /z/, /h/, /m/, /n/, /r/, /w/, /j/
• Vowels: /a/, /e/, /o/, /i/, /u/

All of these vowels and consonants are used in Japanese. Hence, they were all familiar to the current participants^3^.

The participants were also told that Droidese phonology generally requires that a word should consist of three CV syllables (e.g., /ni.so.de/–syllable boundaries indicated by dots). They were asked to use the Japanese *katakana* orthography, in which one letter generally corresponds to one (C)V syllable, to express their responses. The *katakana* system was utilized since this is the orthography that is used to write loanwords and previously unknown words. The participants were also told that Droidese has no words with three identical CV syllables (e.g., /de.de.de/) or those with geminates, long vowels, or consonants with secondary palatalization. The participants were asked to come up with three different names for each stimulus. Thus, nine consonants (i.e., 3 names × 3 syllables) were obtained from each participant for each stimulus.

### Experiment 1

#### Stimulus Movie Production

**FIGURE 3:**
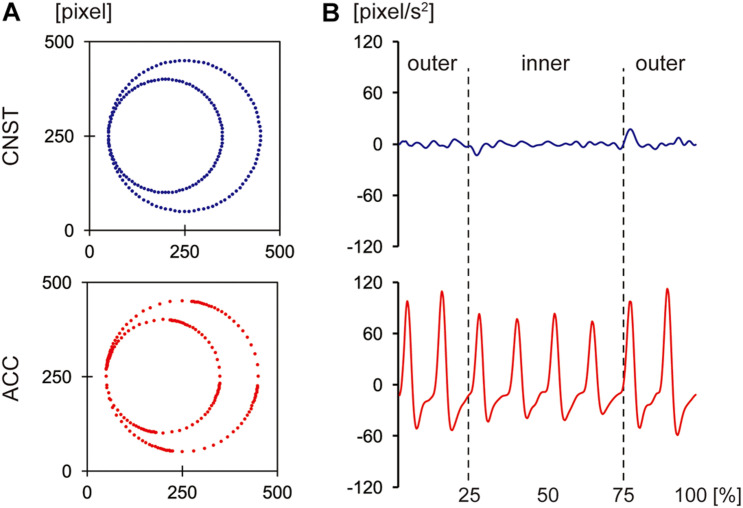
Properties of the visual stimuli: positional changes per given time interval **(A)** and acceleration profiles **(B)** of the point of light for constant velocity motion (CNST, the top panels) and accelerated motion (ACC, the bottom panels). The *x*-axis in **(B)** shows the normalized time course of each motion in percentage.

Experiment 1 used visual motion stimuli with two different kinematic properties: CNST vs. ACC. The movements were presented to the participants as point-light display movies (see Supplementary Videos S1, S2). In the CNST condition, the point of light moved smoothly without tangible change in speed while in the ACC condition, the point of light moved with acceleration and deceleration (see Figure 3B). Of note, these two movements followed the same path (i.e., the outer and inner circular paths, as shown in Figure 3A). The point-light display movies were created using LabVIEW (version 2016 for Windows, National Instruments, United States); the algorithm is described in detail in the Appendix. The draw-area size was 500 × 500 pixels in a black square picture frame. The point of light was a white solid circle with a radius of 8 pixels. The time duration of moving stimuli was 8 s, and was identical between the two motion conditions.

#### Participants

Forty-three (35 males and 8 females, aged 19-24) university students participated in this experiment after providing written consent forms. All participants reported normal or corrected normal vision.

#### Procedure

The stimulus movies were displayed on a screen using an LCD projector and were played back repeatedly without a break. The point of light alternately moved on the outer and inner circular paths. Before the main session, the participants observed both point-light display movies for 15 s to familiarize themselves with the stimuli.

During the main session, the participants performed the free name elicitation task while watching each of the target movies. Within each trial, they were allowed to watch the target movie as many times as they liked until they came up with three Droidese words. They used a booklet with answer boxes in which they wrote three Droidese words for each target movie. The order of the target movies was counterbalanced among the participants.

After the main session, the participants were assigned a drawing task, where they were asked to recall the trajectories of the motions that they watched and to draw their trajectories as accurately as possible. This drawing task was designed so that we could perform a post-hoc analysis of the possible effects of the misperceptions of motion path shapes. As an additional supplementary task, the participants performed a free name elicitation task using Köhler’s static pictures (Figure 1A). Half of the participants responded to the round shape first, while the other half responded to the angular shape first. This supplementary task aimed to test whether we can replicate the shape-sound associations observed in previous studies that used static image stimuli. Experiment 1 lasted for about 30 min in total.

In the drawing task, all of the participants drew a round figure for the CNST motion. However, six participants drew a polygonal figure for the ACC motion, indicating that they may have misperceived the ACC motion as having angular trajectories. In order to examine the possible effects of the misperception, the whole dataset was divided into two sub-groups: The *Round group* (*N* = 37) consisting of the participants who drew a round path and the *Angular group* (*N* = 6) consisting of the participants who drew an angular path.

### Experiment 2

#### Participants

Forty-four (41 males and 3 females, aged 19-21) university students participated in Experiment 2 after providing written consent forms. All of the participants reported that they preferred using their right hand to perform the task. They also reported normal sensorimotor functions of their hands and arms. None of them participated in Experiment 1.

#### Apparatus and Motion Stimulus

**FIGURE 4:**
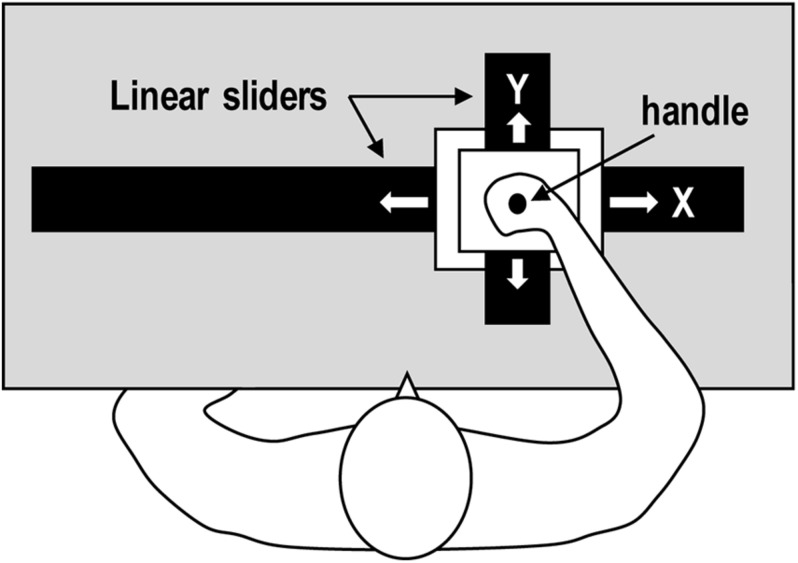
A planar, two-dimensional robotic manipulandum.

The experiment used a planar, two-dimensional, robotic manipulandum that moved the participants’ hands (Figure 4). The planar robotic manipulandum consisted of two orthogonal linear sliders that were separately controlled by a direct-power motor (Sakamoto and Kondo, 2015). A handle on the manipulandum continuously moved along a circular path with two different kinematic properties on a horizontal plane. The handle’s kinematic patterns were similar to those of the point-light display movies used in Experiment 1 (Figure 3, see also Supplementary Videos S3, S4). The algorithm is described in detail in the Appendix.

The participants held the handle of the manipulandum with their right hand while sitting on a chair. The participants were asked not to move their hands, and instead, to let the manipulandum move their hands for them. The participants’ right elbows were kept on the horizontal plane by an arm-bracing apparatus. An opaque acrylic board was mounted horizontally above the manipulandum in order to prevent the participants from directly observing their own arm movement.

#### Procedures

At the beginning of the main task, the participants were informed that the handle would move repeatedly along a path with the same shape on the two-dimensional, horizontal plane, but that it would do so with different kinematic properties. Then, they felt each type of movement (CNST and ACC) for approximately 15 s. Once each participant confirmed that he or she could feel the difference between the two kinematic patterns, the name elicitation task began.

During the main session, the participants verbally reported three Droidese words for each target motion. An experimenter recorded their responses. The order of the target motions was counterbalanced between the participants to control for order effects. Within each trial, the participants were allowed to feel the target motion as many times as they liked until they came up with three words.

After completing the main session, the participants were asked to recall and draw the shapes of the paths that they felt as accurately as possible. In addition, they performed the name elicitation task for Köhler’s figures (Figure 1A). The order of the target figures was counterbalanced among the participants. Experiment 2 lasted for about 20 min in total.

During the drawing task, all of the participants drew a round figure for the CNST motion. However, for the ACC motion, 25 participants drew a round figure, whereas 19 participants drew a polygonal figure, indicating that they have mistakenly felt angular motion paths (recall that all of the stimuli followed a round path). The whole dataset was divided into two sub-groups: The *Round group* (*N* = 25) consisting of the participants who drew a round path and the *Angular group* (*N* = 19) consisting of the participants who drew an angular path.

### Statistical Analysis

The data analysis was carried out according to the procedure by Shinohara et al. (2016). The nine consonants obtained from each participant for each stimulus were used to compute two dependent variables, that is, the proportions (*P*_*ij*_) of the obstruents (/p/, /t/, /k/, /b/, /d/, /g/, /s/, /z/) and the sonorants (/m/, /n/, /r/, /w/, /j/) to the total nine consonants:^4^

(1) $$P_{i\,j}=f_{i\,j}/n$$

where *f*_*ij*_ is the number of obstruents (or sonorants) produced by a participant *i* and a stimulus *j*, and *n* = 9. To make these proportions more suitable for ANOVA, arcsine transformation was applied using the following equation (Mori and Yoshida, 1990):

(2) $$X_{i\,j}=\sin^{-1}\sqrt{P_{i\,j}}$$

If *P*_*ij*_ is 1 or 0, they were adjusted and replaced with (*n* – 0.25)/*n* and 0.25/*n*, respectively (Mori and Yoshida, 1990).

For statistical analyses involving the two dependent variables, a one-way repeated-measures ANOVA with motion stimuli (CNST vs. ACC) as the within-participants factor was performed separately for the datasets from Experiments 1 and 2. For the dataset from Experiment 2, a two-way, mixed-design ANOVA with motion stimuli as the within-participants factor and sub-groups (Round group vs. Angular group) as the between-participants factor was conducted in order to examine the possible effects of the misperceptions of motion path shapes^5^. If the effect of the misperceptions did not reach significance, we further performed a two-way, mixed-design ANOVA with motion stimuli as the within-participants factor and modality (vision vs. proprioception) as the between-participants factor. This analysis tested whether the sound symbolic connection at issue would hold for both visual inputs (Experiment 1) and proprioceptive inputs (Experiment 2).

Finally, for the measurements obtained from the additional naming task of static figures, a one-way repeated measures ANOVA with the static image difference (*maluma* vs. *takete*) as the within-participants factor was performed separately for the datasets from Experiments 1 and 2. Furthermore, a two-way, mixed-design ANOVA with static image stimuli as the within-participants factor and experiment group (Experiment 1 vs. Experiment 2) as the between-participants factor was conducted. These analyses aimed to test whether we can replicate the shape-sound associations that were observed from previous studies using static image stimuli.

For all ANOVA tests, partial eta-squared (η_*p*_^2^) was calculated as a measure of effect size. All statistics were computed using SPSS (version 24 for MacOS, IBM, United States).

## Results

### Sound Selection for Motion Stimuli

**FIGURE 5:**
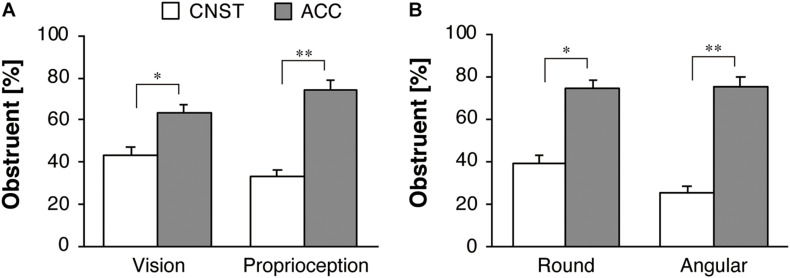
Response percentages of obstruents for motion stimuli for the whole dataset: **(A)** vision in Experiment 1 and proprioception in Experiment 2, **(B)** the two sub-groups in Experiment 2. The white and gray bars represent the average proportions for constant velocity motion (CNST) and accelerated motion (ACC), respectively. The error bars represent standard errors, *p < 0.05, **p < 0.01.

Figure 5A shows the group mean and standard errors (SE) of the proportions [*P*_*ij*_ in Eq. (1)] of obstruent responses for the motion stimuli (CNST vs. ACC). In the separate datasets from Experiments 1 and 2, a significant effect of motion stimuli was found for the visual condition [*F*(1,42) = 7.39, *p* < 0.05, η_*p*_^2^ = 0.15] and proprioceptive condition [*F*(1,43) = 25.50, *p* < 0.001, η_*p*_^2^ = 0.37]. The participants used more obstruents for the ACC motion than for the CNST motion to a significant degree.

Figure 5B illustrates the percent obstruent responses, broken down by the two sub-groups in Experiment 2. Recall that one group consisted of the participants who (correctly) drew a round path after the main task (Round group), while the other consisted of the participants who (mistakenly) drew an angular path after the main task (Angular group). A significant effect of the motion factor (CNST vs. ACC) was found [*F*(1,42) = 27.54, *p* < 0.001, η_*p*_^2^ = 0.40]. Neither the main effect of the sub-group (Round vs. Angular groups) [*F*(1,42) = 1.28, *p* = 0.27, η_*p*_^2^ = 0.03] nor the interaction between the sub-group factor and the motion factor [*F*(1,42) = 1.95, *p* = 0.17, η_*p*_^2^ = 0.04] was significant. A one-way repeated-measures ANOVA with the motion factor was performed separately for each of the two sub-groups. A significant effect of the motion factor was found both for the Round group [*F*(1,24) = 7.60, *p* < 0.05, η_*p*_^2^ = 0.24] and the Angular group [*F*(1,18) = 23.50, *p* < 0.001, η_*p*_^2^ = 0.57], but the effect size of motion factor was larger for the Angular group than for the Round group.

**FIGURE 6:**
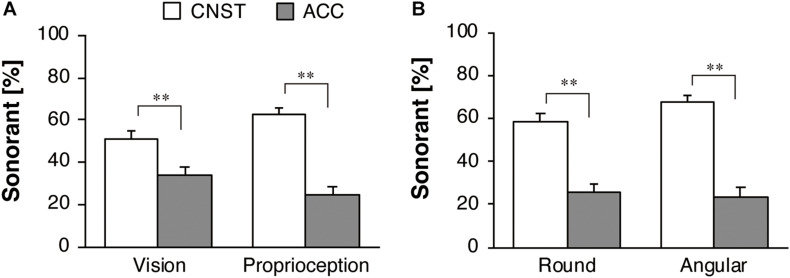
Response percentages of sonorants for motion stimuli for the whole dataset: **(A)** vision in Experiment 1 and proprioception in Experiment 2, **(B)** the two sub-groups in Experiment 2. The white and gray bars represent the average proportions for constant velocity motion (CNST) and accelerated motion (ACC), respectively. The error bars represent standard errors, **p < 0.01.

Figure 6A shows the group mean and SE of the proportions of sonorant responses for the motion stimuli. In the separate datasets from Experiments 1 and 2, a significant effect of motion stimuli was found for the visual condition [*F*(1,42) = 15.18, *p* < 0.001, η_*p*_^2^ = 0.27] and proprioceptive condition [*F*(1,43) = 41.97, *p* < 0.001, η_*p*_^2^ = 0.49]. In contrast to the obstruents, as mentioned above, the participants used more sonorants for the CNST motion than for the ACC motion to a significant degree.

Figure 6B illustrates the percent of sonorant responses, broken down by the two sub-groups in Experiment 2. A significant effect of motion factor was found [*F*(1,42) = 41.30, *p* < 0.001, η_*p*_^2^ = 0.50]. There was no significant main effect of the sub-group [*F*(1,42) = 0.02, *p* = 0.89, η_*p*_^2^ = 0.001] and the interaction between the two factors [*F*(1,42) = 0.23, *p* = 0.63, η_*p*_^2^ = 0.01]. Thus, a one-way repeated-measures ANOVA with the motion factor was performed separately for each of the two sub-groups. A significant effect of the motion factor was found for the Round group [*F*(1,24) = 19.58, *p* < 0.001, η_*p*_^2^ = 0.45] and the Angular group [*F*(1,18) = 22.33, *p* < 0.001, η_*p*_^2^ = 0.55]. Both the sub-groups showed larger, similar effect size of motion factor.

These results support our hypothesis that obstruents are more likely to be associated with ACC motions than with CNST motions, whereas sonorants are more likely to be associated with CNST motions than with ACC motions.

### Effect of Modality for Motion Stimuli (Vision vs. Proprioception)

For obstruent selection, the comparison between the results of Experiment 1 and those of Experiment 2 shows that modality had no significant effect [*F*(1,85) = 0.44, *p* = 0.51, η_*p*_^2^ = 0.005], while motion factor significantly affected the obstruent responses [*F*(1,85) = 31.76, *p* < 0.001, η_*p*_^2^ = 0.27]. The interaction between the two factors (modality and motion conditions) was statistically significant, but this effect was weak [*F*(1,85) = 5.24, *p* < 0.05, η_*p*_^2^ = 0.06]. For sonorant selection, modality had no significant effect [*F*(1,85) = 0.19, *p* = 0.66, η_*p*_^2^ = 0.002], while motion factor significantly affected the sonorant responses [*F*(1,85) = 56.14, *p* < 0.001, η_*p*_^2^ = 0.40]. The interaction between the two factors was statistically significant, but this effect was weak [*F*(1,85) = 7.89, *p* < 0.01, η_*p*_^2^ = 0.09].

In order to examine the responses to the motion stimuli obtained from the participants who correctly perceived the ACC path shapes, an additional comparison was performed between the Round group dataset in Experiment 1 and the Round group dataset in Experiment 2. For obstruent selection, a significant effect of motion factor was found [*F*(1,60) = 14.06, *p* < 0.001, η_*p*_^2^ = 0.19]. Neither the main effect of the modality [*F*(1,60) = 0.00, *p* = 0.97, η_*p*_^2^ = 0.00] nor the interaction between the two factors [*F*(1,60) = 1.70, *p* = 0.20, η_*p*_^2^ = 0.03] was significant. For sonorant selection, a significant effect of motion factor was found [*F*(1,60) = 34.19, *p* < 0.001, η_*p*_^2^ = 0.36], while modality had no significant effect [*F*(1,60) = 0.35, *p* = 0.56, η_*p*_^2^ = 0.01]. The interaction between the two factors was statistically significant, but this effect was weak [*F*(1,60) = 6.5, *p* < 0.05, η_*p*_^2^ = 0.10].

To summarize, the hypothesized sound-symbolic effects, i.e., the associations between obstruents and ACC and between sonorants and CNST, were detected in both of the modalities: vision and proprioception. The effect sizes did not differ between the two modality conditions, particularly if the participants correctly perceived round motion paths for the ACC stimuli.

### Sound Selection for Static Figures

**FIGURE 7:**
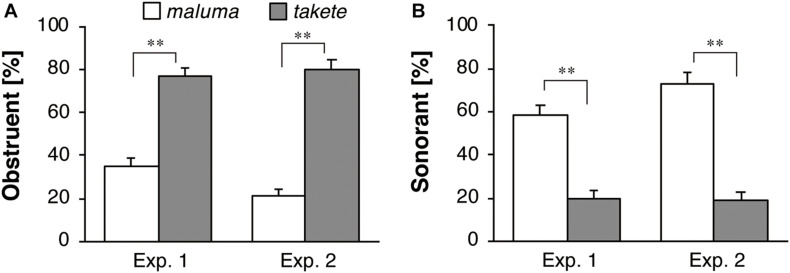
Response percentages of **(A)** obstruents and **(B)** sonorants for Köhler’s static figure stimuli. The white and gray bars represent the average proportions for the *maluma* and the *takete* figures, respectively. The error bars represent standard errors, **p < 0.01.

Figure 7 shows the results of the follow-up task in which the participants were asked to come up with names for the static shapes. Figures 7A,B describe the results for the obstruent and sonorant responses, respectively. This task was conducted in order to ensure that the current participants showed responses that were similar to those of the participants who were tested in the previous studies using static image stimuli. For obstruent selection, a significant effect of the figure shape was found for the whole datasets of both Experiment 1 [*F*(1,42) = 20.95, *p* < 0.001, η_*p*_^2^ = 0.33] and Experiment 2 [*F*(1,43) = 47.14, *p* < 0.001, η_*p*_^2^ = 0.52]. The results show that obstruents were more likely to be used for the static angular *takete* shape than for the static round *maluma* shape. For sonorant selection, a significant effect of figure shape was found in Experiment 1 [*F*(1,42) = 86.18, *p* < 0.001, η_*p*_^2^ = 0.67] and Experiment 2 [*F*(1,43) = 84.95, *p* < 0.001, η_*p*_^2^ = 0.66]. The results show that sonorants were more likely to be used for the static round *maluma* shape than for the static angular *takete* shape.

A two-way ANOVA for the obstruent responses revealed no significant effects of the participant group factor (Experiment 1 vs. Experiment 2) [*F*(1,85) = 1.83, *p* = 0.18, η_*p*_^2^ = 0.02], while there was a significant effect of static figure shape (*maluma* vs. *takete*) [*F*(1,85) = 65.85, *p* < 0.001, η_*p*_^2^ = 0.44]. No significant interaction between these two factors, i.e., participant group and shape, was found [*F*(1,85) = 3.11, *p* = 0.08, η_*p*_^2^ = 0.04]. For sonorant selection, the participant group factor had no significant effects [*F*(1,85) = 0.12, *p* = 0.74, η_*p*_^2^ = 0.001], while a significant effect of static figure shape was noted [*F*(1,85) = 168.63, *p* < 0.001, η_*p*_^2^ = 0.67]. There was no significant interaction between the two factors [*F*(1,85) = 1.46, *p* = 0.23, η_*p*_^2^ = 0.02].

The comparison in obstruent selection between the Round group dataset in Experiment 1 and the Round group dataset in Experiment 2 revealed a significant effect of the static figure shape [*F*(1,60) = 39.80, *p* < 0.001, η_*p*_^2^ = 0.40], while the participant group factor had no significant effect [*F*(1,60) = 1.43, *p* = 0.24, η_*p*_^2^ = 0.02]. Neither was the interaction between these two factors significant [*F*(1,60) = 3.49, *p* = 0.07, η_*p*_^2^ = 0.06]. For sonorant selection, a significant effect of the static figure shape was observed [*F*(1,60) = 108.40, *p* < 0.001, η_*p*_^2^ = 0.64], while the participant group factor had no significant effect [*F*(1,60) = 1.18, *p* = 0.28, η_*p*_^2^ = 0.02]. The interaction between the two factors was not significant [*F*(1,60) = 1.30, *p* = 0.26, η_*p*_^2^ = 0.02].

To summarize, the data from the present participants show that obstruents are more likely to be associated with the angular static shape than with the round static shape, whereas sonorants are more likely to be associated with the round static shape than with the angular static shape.

## Discussion

### Effects of Kinematic Properties of Motion on Sound Symbolism

The current experiments addressed the issue of whether a particular aspect of dynamic movement–presence or absence of noticeable acceleration–can be associated with certain types of sounds. Care was taken to explore the effects of acceleration as distinct from static shapes. The results show that motions with clear acceleration and deceleration tend to be associated with obstruents, whereas motions that do not involve such abrupt changes in speed are likely to be associated with sonorants. Since the trajectories of the stimuli are constant across the two conditions in both Experiments 1 and 2, the results suggest that there can be a cross-modal correspondence between different acceleration profiles and different classes of sounds, in addition to the well-known shape-sound associations (Köhler, 1929, 1947).

The two experiments reported in this paper demonstrated that sound symbolic associations hold for both the visual and the proprioceptive properties of motions. By holding the movement trajectories constant and varying the acceleration profiles of the movements, we made a distinction between the effects of motion path shape and acceleration, the latter of which is a *kinematic* property of movement. The results show that obstruents are more likely to be associated with motions that involve acceleration or deceleration, whereas sonorants tend to be associated with smooth motions with constant speed. Notably, the current participants provided responses that were similar to those of the participants who were tested in previous related experiments on “static shape”-sound symbolism. The factor of participant selections does not appear to affect the skewed consonant distribution to the motion stimuli. Therefore, the overall results suggest that there exists certain association between a particular kinematic properties and linguistic sounds. Moreover, Experiment 2 showed that this association held when the participants felt the movement without visual stimuli. We believe that this type of cross-modal association is worth pursuing at a more in-depth perspective, as it could tell us more about the bodily basis of sound symbolic associations.

In addition to these general tendencies of the kinematic properties-linguistic sound associations, the present study observed that ca. 45% of the participants felt angular motion paths for the accelerated/decelerated passive movements without vision (i.e., Angular group in Experiment 2). The effect size of the motion factor, constant vs. acceleration/deceleration, was larger for the Angular group than for the Round group in obstruent selection, but did not differ in sonorant selection. Although the misperception of motion paths might have resulted in stronger obstruent-acceleration associations, the Round group also showed significant skewed selection of obstruents. Thus, it seems safe to conclude that acceleration is a significant cue to evoke larger skewed selection of obstruents, particularly in proprioceptive perception. Since it is not clear why acceleration could cause some participants to perceive erroneously angular paths of motion, in-depth future study is expected.

### Origins of Motion-Sound Symbolism

How did certain consonants come to be associated with particular kinematic features of motion stimuli independent of the shape of the motion path? Sidhu and Pexman (2017) suggested five possible mechanisms for sound symbolism (see also Spence, 2011 for cross-modal correspondences): (a) phonetic/phonological features statistically co-occurring with stimuli in the environment, (b) shared properties among phonetic features and stimuli, (c) overlapping neural processes, (d) associations developed through the evolutional process, and (e) language-specific patterns. Some of these mechanisms appear to be suitable in accounting for the kinematics-sound symbolic associations that were identified in the current study.

As for the first mechanism proposed by Sidhu and Pexman (2017), i.e., phonetic features statistically co-occurring with stimuli in the environment, Changizi (2011) stated that phonemes in speech have similarities with frequently occurring sounds in nature. For instance, the sounds that solid objects make when they hit each other or when one object slides on the other may be analogous to plosives and fricatives, respectively. Similar analogy may hold between the sounds that accompany bodily actions, such as hitting or sliding an object using a tool, and linguistic sounds, such as plosives and fricatives. In these cases, the sounds produced by the actions and the associated linguistic sounds share some phonetic similarities. In fact, the sounds accompanying an action is caused by physical dynamics in the action that involve quantities such as force, mass/inertia, velocity, and acceleration/deceleration. That is, the physical properties of sounds that accompany movements and actions are determined by such physical quantities. The actions involving acute acceleration/deceleration patterns are more likely to be associated with plosives or fricatives (i.e., obstruents) than sonorants because such actions arguably tend to produce physical sounds that share similar phonetic properties with obstruents rather than sonorants; for example, hitting a hard surface with a stick usually produces aperiodic sounds rather than periodic sounds, which are, acoustically speaking, more like obstruents than sonorants. This could raise a possibility that the sound-symbolic associations between patterns of acceleration/deceleration and consonants can be the potential driving force, both for shape-sound symbolism and motion-sound symbolism (though cf. Margiotoudi and Pulvermüller, 2020). Consider the patterns of acceleration described in Figure 2, where the trajectory of hand gesture tracing a round figure has a more or less constant speed, while the trajectory of hand gesture tracing an angular figure shows clear acceleration and deceleration. The well-known shape-sound symbolism that associates angular figures with obstruents, such as plosives and fricatives, can be explained by the above-mentioned mechanism. In the same way, the correlation between action sounds and action kinematics may be one of the motivations supporting motion-sound symbolism. The results from the two experiments reported here are consistent with this hypothesis: the phonetic features of the consonants occurring in the elicited words tended to share certain properties that were typical for the types of motion in the stimuli. That is, the patters of acceleration/deceleration in the motion were consistent with the associated categories of consonants in the elicited words, considering the kinematic explanation above. Further investigation of the associations between the sounds produced by bodily actions and kinematic properties of action, as well as the relationships between speech sounds and action sounds, would provide stronger evidence for this inference.

The mechanism of shared properties among phonetic features and stimuli may also account for the kinematics-sound symbolic associations. Stops and affricates require the occlusion of the vocal tract followed by an abrupt release and, thus, involve a cessation of the oral airflow. By contrast, sonorants are produced with less abrupt changes in the motion of the vocal organs and involve no obstruction of the airflow. The present results, particularly those from Experiment 2, may be compatible with the shared properties hypothesis (Ramachandran and Hubbard, 2001; Vainio et al., 2013, 2017). That is, stops and affricatives, during which oral airflow is trapped once in the oral cavity and then released, were associated with the motion stimuli consisting of deceleration, stop, and acceleration phases, whereas sonorants without complete stoppages of the airflows were associated with the constant motion stimuli that did not include any stop phases. Thus, the shared properties hypothesis suggests the iconic relationship between the obstruent-sonorant distinction and the acceleration patterns.

Some theories propose that the sound-symbolic associations themselves are a consequence of evolution. Nielsen and Rendall (2011, 2013) theorized that the evolved semantic-affective associates of two types of sounds, i.e., harsh, noisy, and punctuate sounds vs. smoother, more harmonic sounds, may extend to phonemes with similar acoustic properties: obstruents and sonorants. They stated that for instance, many primates and other animal species use the former sounds in situations of hostility, aggression, and high arousal, and the latter sounds in situations of positive affiliation, contact, and low arousal (Owren and Rendall, 2001). The semantic-affective associates of these two types of sounds are also evident in humans (Rendall, 2003). In the natural world, life-threatening situations require preparatory quick motor responses, such as fight or flight at high arousal level, whereas safe situations do not require such a quick motor response. Biomechanically, the quick motor responses demand a larger power that can explosively accelerate the whole body. Thus, the evolved semantic-affective association mechanism could also contribute to associations between obstruents/sonorants phonemes and accelerated/smooth motions. In short, various mechanisms may underlie the motion-sound symbolic connections that are identified in the current experiment.

### Limitations and Issues to Be Investigated

As we have stated in the introduction, we have only started to understand whether and how dynamic movements can be symbolically represented in natural languages. There are several remaining issues that can be explored in future research. First, the participants’ hands were passively moved by a manipulandum in Experiment 2 because we needed to precisely control the motion trajectory and kinematic pattern. Thus, the present results leave unanswered the question of whether people are able to map their motor commands (or motor representation in a cognitive level) onto particular sets of linguistic sounds. The use of experimental conditions including voluntary movements (e.g., Bernardis and Gentilucci, 2006) and their kinematics analyses would help address this issue.

The present outcomes lend support to the conclusion that the motion-kinematics:word-sound matching bias is a bona fide effect. The current results suggest that the observed effects are not attributable to a specific set of experimenter-selected words, thereby overcoming a potential methodological limitation pointed out by Westbury (2005). One may quibble, however, that the present results depend on the substantial differences in the acceleration parameter that we chose for the current stimuli (see Appendix). More work is warranted to test how participant biases are sensitive to such specific details of acceleration magnitudes. In this regard, the novel motion stimuli generation techniques introduced in this paper offer considerable promise for future studies because they allow for a virtually limitless number, range, and variety of controlled motions to be generated for future testing.

Another limitation of the present study is that we targeted only Japanese native speakers. While studies of sound symbolism tend to focus on the universality of sound symbolic patterns, we do observe non-trivial cross-linguistic differences (Saji et al., 2013). The current experiment should thus be ideally replicated with speakers of other languages. Further, we assumed throughout this paper that speakers possess “linguistic knowledge,” broadly construed, which allows them to produce/perceive sounds as well as to create new names; this is what allows speakers to utter and understand novel utterances, i.e., utterances that they have not heard before in their lives. Even though we neither presented physical sounds as stimuli nor analyzed the sounds that the participants produced in their responses, it seems safe to assume that our experiment tapped this body of knowledge, and in this very sense, our experiments allow us to make conclusions about linguistic sounds (i.e. linguistic knowledge). We believe that this is a rather uncontroversial assumption to make in sound symbolic–or more broadly, cognitive linguistic–research; recall that our method follows that of Berlin (2006) and Shinohara et al. (2016), among many others. However, to the extent that this assumption should be reexamined, the current experiment can be followed-up on using different tasks with different types of stimuli (e.g. auditory stimuli). See e.g., Sidhu et al. (2016), Cuskley et al. (2017), and Westbury et al. (2018) on recent discussion on potential task effects in sound symbolic research, including the effects of using auditory/orthographic stimuli.

Finally, more experimentation is necessary to bear upon the issues briefly raised in the introduction: how motion-related sound symbolism contributed to the origin and development of human languages (Larsson, 2015) and how they contribute to the acquisition of first languages (Imai and Kita, 2014). A natural question that arises in this context is whether we are able to replicate the current findings with young children and/or toddlers. Thus, the current study opens up new opportunities for future exploration in this ever-growing body of cross-modal perception research.

## Conclusion

Despite the fact that the relationship between sounds and meanings is generally arbitrary (de Saussure, 1916), we now have a substantial body of evidence proving that sounds themselves can have meanings. Most studies on sound symbolism used semantic dimensions, including a large/small distinction and an angular/round distinction, while other studies used static visual images. We expanded the previous body of literature, following a few important precedents (Cuskley, 2013; Koppensteiner et al., 2016; Shinohara et al., 2016), that dynamic motions can lead to sound symbolic associations. The present approach provides the first evidence suggesting that some types of consonants are associated with acceleration profiles of motion stimuli in multimodal perceptual domains, at the very least, vision and proprioception.

In conclusion, the present study has added an additional piece of evidence demonstrating that bodily action-based information, in particular proprioceptive information, could lead to sound symbolic patterns.

## Data Availability Statement

The raw data supporting the conclusions of this article will be made available by the authors, without undue reservation.

## Ethics Statement

The studies involving human participants were reviewed and approved by the Ethics review board of Tokyo University of Agriculture and Technology (Permit No. 27-04). The patients/participants provided their written informed consent to participate in this study.

## Author Contributions

HT conceived of and designed the study. KS implemented the design for Experiment 1. SK implemented the design for Experiment 2. HT and KS collected and analyzed the data and prepared the figures. HT, KS, and SK wrote the manuscript. All authors contributed to the article and approved the submitted version.

## Conflict of Interest

The authors declare that the research was conducted in the absence of any commercial or financial relationships that could be construed as a potential conflict of interest.

## Footnotes

1 Research on sound symbolism has been flourishing in linguistics, cognitive science, neuroscience, psychology, and many other related fields; as such, a number of informative review articles are now available (Akita, 2009, 2015; Dingemanse et al., 2015; Lacey et al., 2016; Sidhu and Pexman, 2017; Itagaki et al., 2019).

2 From a mechanics point of view, it is impossible to keep the speed constant when an object is moving on an angular path, because such movement necessarily involves acceleration and deceleration while it is passing through an angular point. From this reason, we avoided using an angular path, and selected only a round path.

3 Note that the Japanese sound of /r/ is flap, which is different from English /r/ or /l/.

4 We set aside /h/ from the count of target sounds as the numerator in Eq. (1) because whether /h/ should be treated as an obstruent or a sonorant remains controversial. Jakobson et al. (1963) and Chomsky and Halle (1968) classified /h/ as a voiceless glide (i.e., a sonorants), while Katamba (1989) classified it as an obstruent. According to the current convention in the IPA system (International Phonetic Association, 2015), /h/ is classified as a voiceless fricative (i.e., an obstruent).

5 This breakdown analysis was not carried out in the dataset from Experiment 1 because the sample size for the Angular group was too small (*N* = 6).

## Supplementary Material

Supplementary Tables 1–3 Descriptive Statistics Tables.

Supplementary Video 1 CNST motion stimulus for visual perception (Experiment 1).

Supplementary Video 2 ACC motion stimulus for visual perception (Experiment 1).

Supplementary Video 3 CNST motion stimulus for proprioceptive perception (Experiment 2).

Supplementary Video 4 ACC motion stimulus for proprioceptive perception (Experiment 2).

## Appendix

### Algorithm to Produce Motion Stimuli

A horizontal position (*x*_*t*_) and a perpendicular position (*y*_*t*_) of the center of the object (i.e., a point of light in Experiment 1 and a handle in Experiment 2) on a motion path at time *t* were defined using the following formulas:

(3) $$x_{t}=r\,\cos\omega_{t}$$

(4) $$y_{t}=r\,\sin\omega_{t}$$

where *r* was the radius of the circular path.

For a quadrant of the circular path, a central angle of ω_*t*_ at time *t* was defined as follows:

(5) $$\omega_{t}=\frac{\pi }{2}\,\left(t+c\right),c=0,1,2,3$$

for the constant velocity motion (CNST)

(6) $$\omega_{t}=\frac{\pi }{2}\,\left(1-a^{25\,t}+c\right),c=0,1,2,3$$

for the accelerated motion (ACC)

where *c* was the number of a quadrant of a circular path before the object moved, *a* was the control parameter of acceleration, and *t* changed, ranging from 0 to 1 [s].

In Experiment 1, *r* = 200 and 150 [pixels] were set for the outer and inner paths, respectively. In Experiment 2, *r* = 80 and 60 [mm] were set for the outer and inner paths, respectively. To produce two stimulus motions, eight quadrant paths in total for both the outer and inner circles were combined and arranged in space in such a way that the left end of the outer and inner ones coincided with each other. In the produced stimuli, the object started moving clockwise from the right end on the outer circle, headed from the left end to the inner circle, went around on the inner circular path, and then returned to the starting point on the outer circle. Each of the two stimulus motions were 4 s long, resulting in 8-s-long stimuli, i.e., 1 [s] × 4 quadrants × 2 circles. The present study used *a* = 0.9 because in a preliminary experiment, we confirmed that the participants (*N* = 3) did not misperceive the ACC motion as having angular trajectories.
